# Supplementary figures and images for: DNA double strand break position leads to distinct gene expression changes and regulates VSG switching pathway choice
Source: PLoS Pathog. 2021 Nov 12;17(11):e1010038. doi: 10.1371/journal.ppat.1010038 (PMC8612549; doi:10.1371/journal.ppat.1010038)

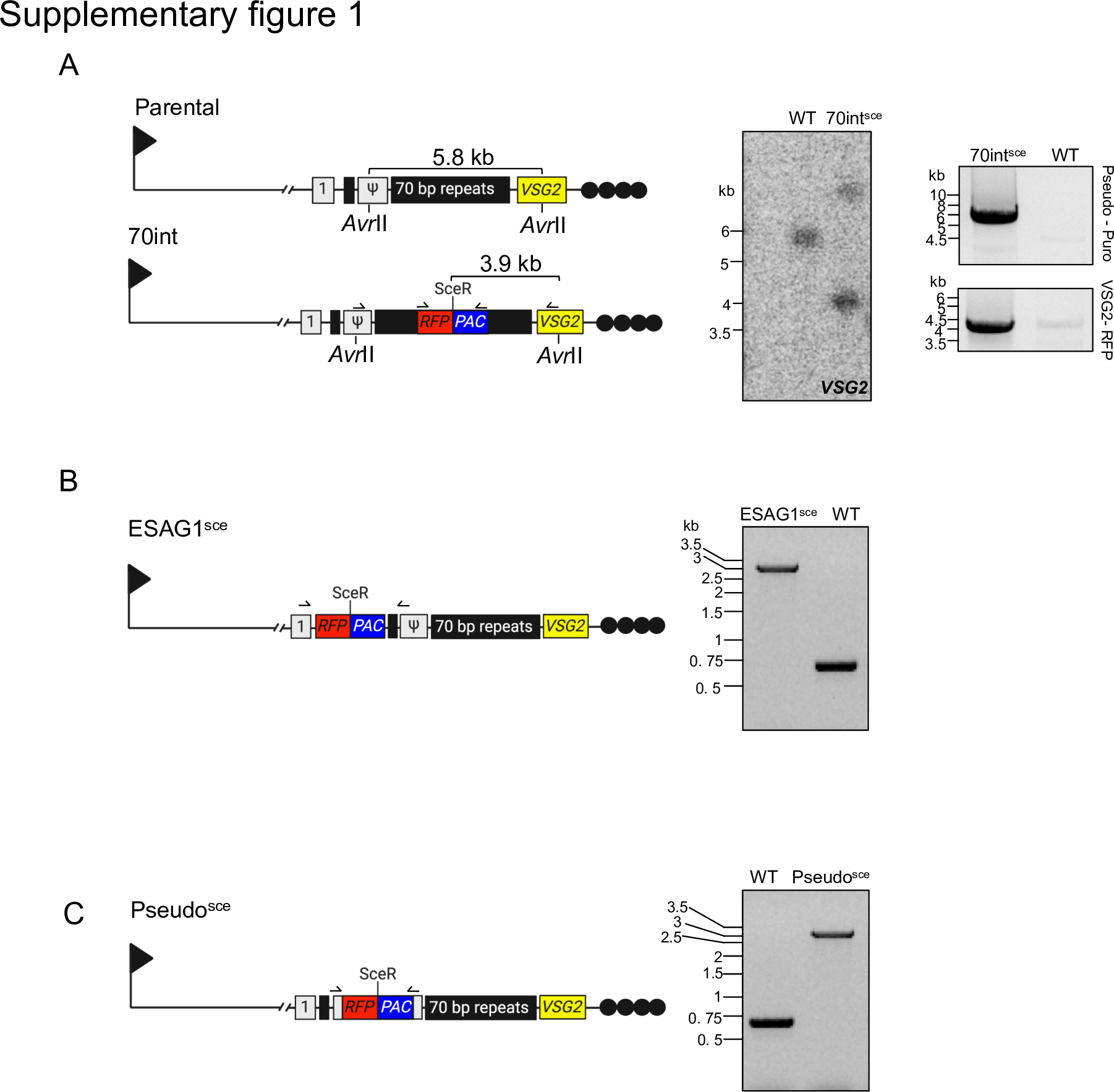

Supplement: S1 Fig — (A) Left panel: Schematic showing the 70intsce cell line. Middle panel: Southern blot. Right panel: PCR assay, correct integration should give a band of approximately 5000 bp using primers from the pseudo gene to PAC and a 4523 bp band from VSG2 to RFP. Relevant restriction sites shown. (B) Left panel: Schematic showing the ESAG1sce cell line. Right panel: PCR assay, correct integration should give a 2334 bp band using primers from ESAG1 to PAC. (C) Left panel: Schematic showing the Pseudosce cell line. Right panel: PCR assay, wild-type should give a band at 600 bp and correct integration of the RFP:PAC cassette should give 2564 bp. Black arrow, BES RNA Pol1 promoter; grey boxes, ESAGS; black boxes, 70 bp repeats; yellow box, VSG; black circles, telomere. RFP:PAC, Red Fluorescent Protein and Puromycin N–Actyltransferase. Line arrow, primer binding sites. (TIF) [file ppat.1010038.s001.tif]

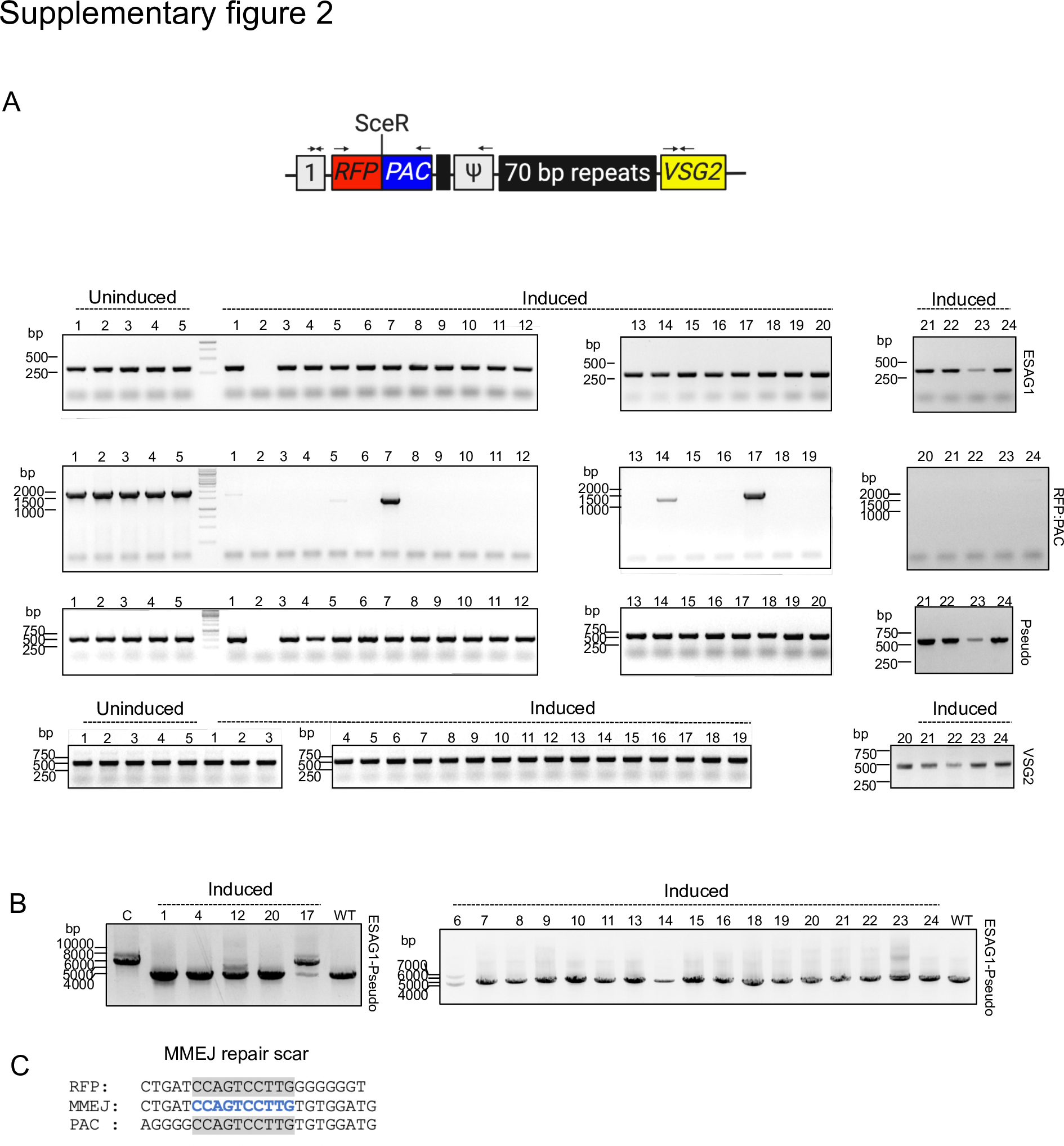

Supplement: S2 Fig — (A) Schematic indicates the location of the primers used. (B) The PCR assays show the presence or absence of ESAG1, Pseudo, RFP—PAC and VSG2 following an I-SceI induced DSB. (C) MMEJ repair scar seen in 13 clones. (TIF) [file ppat.1010038.s002.tif]

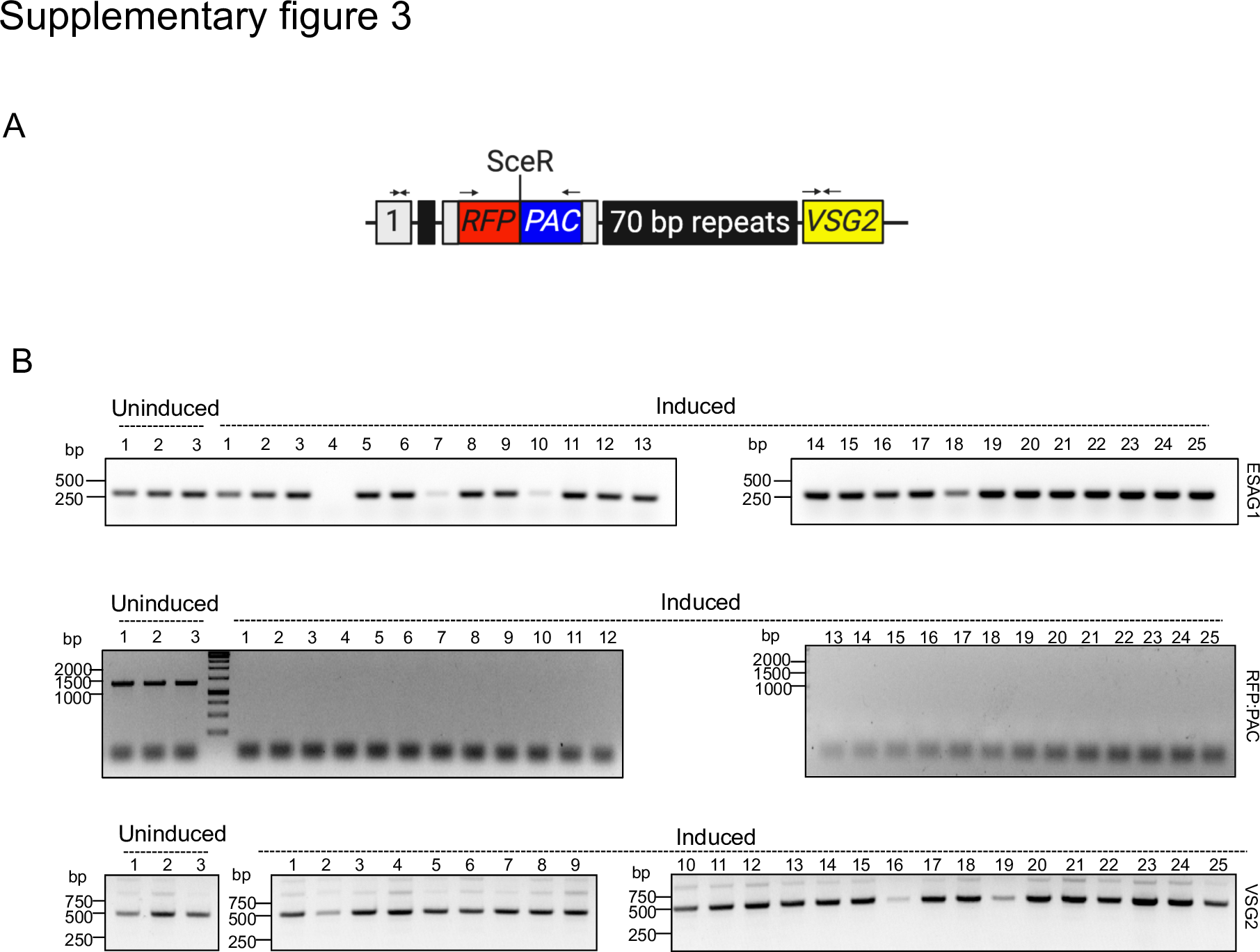

Supplement: S3 Fig — (A) Schematic indicates the location of the primers used. (B) The PCR assays show the presence or absence of ESAG1, RFP:PAC and VSG2 following an I-SceI induced DSB. (TIF) [file ppat.1010038.s003.tif]

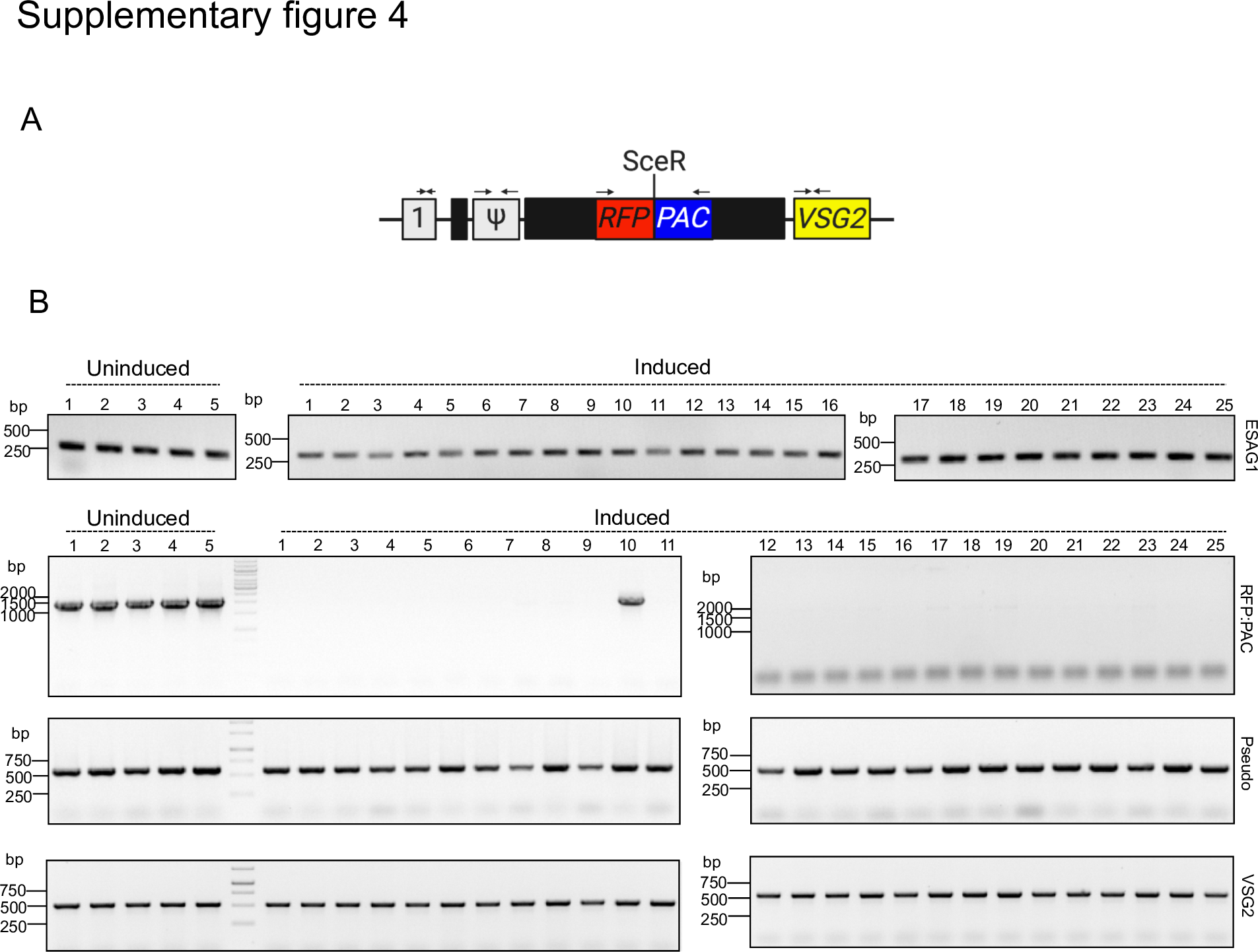

Supplement: S4 Fig — (A) Schematic indicates the location of the primers used. (B) The PCR assays show the presence or absence of ESAG1, Pseudo, RFP:PAC and VSG2 following an I-SceI induced DSB. (TIF) [file ppat.1010038.s004.tif]

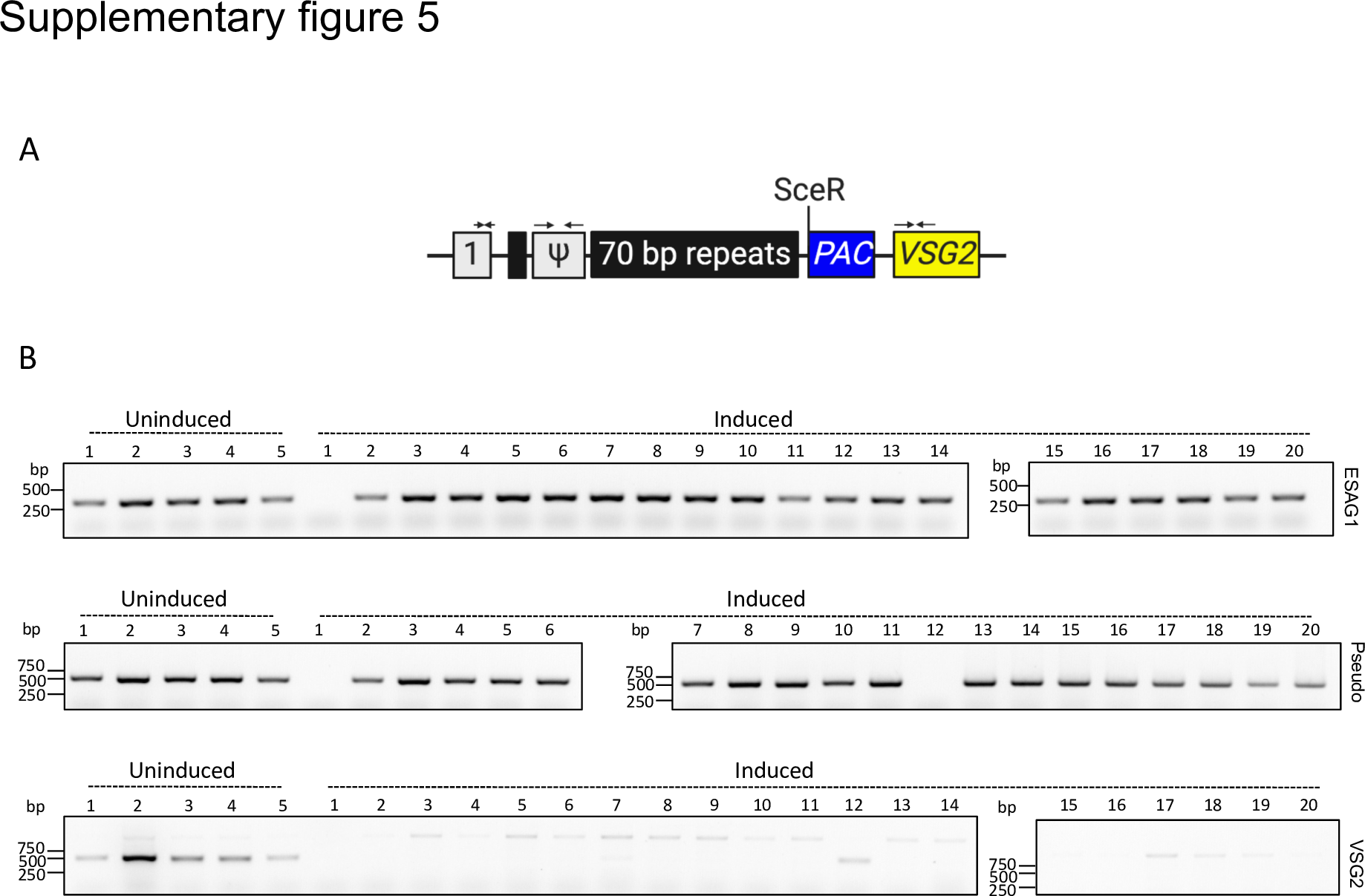

Supplement: S5 Fig — (A) Schematic indicates the location of the primers used. (B) The PCR assays show the presence or absence of ESAG1, Pseudo and VSG2 following an I-SceI induced DSB. (TIF) [file ppat.1010038.s005.tif]

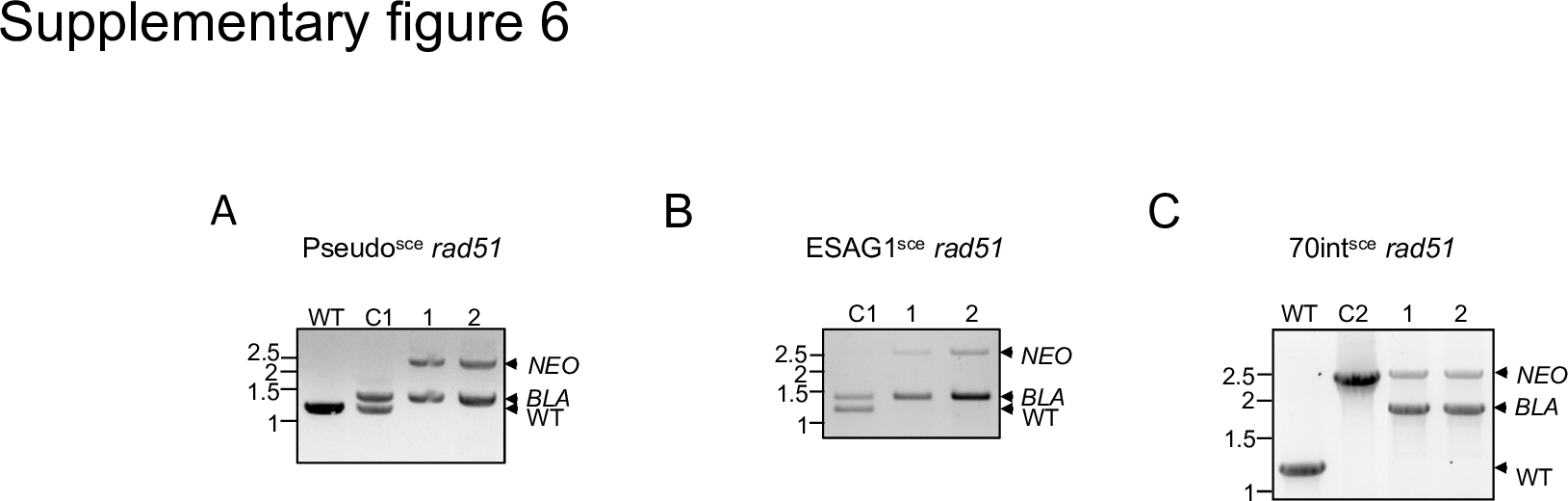

Supplement: S6 Fig — (A) PCR assay confirming rad51 double allele replacement Pseudosce. (B) PCR assay confirming rad51 double allele replacement ESAG1sce. (C) PCR assay confirming rad51 double allele replacement 70intsce. NEO, Neomycin Phosphotransferase; BLA, Blasticidin deaminase. C1, control plasmid for BLA; C2, control plasmid for NEO. (TIF) [file ppat.1010038.s006.tif]

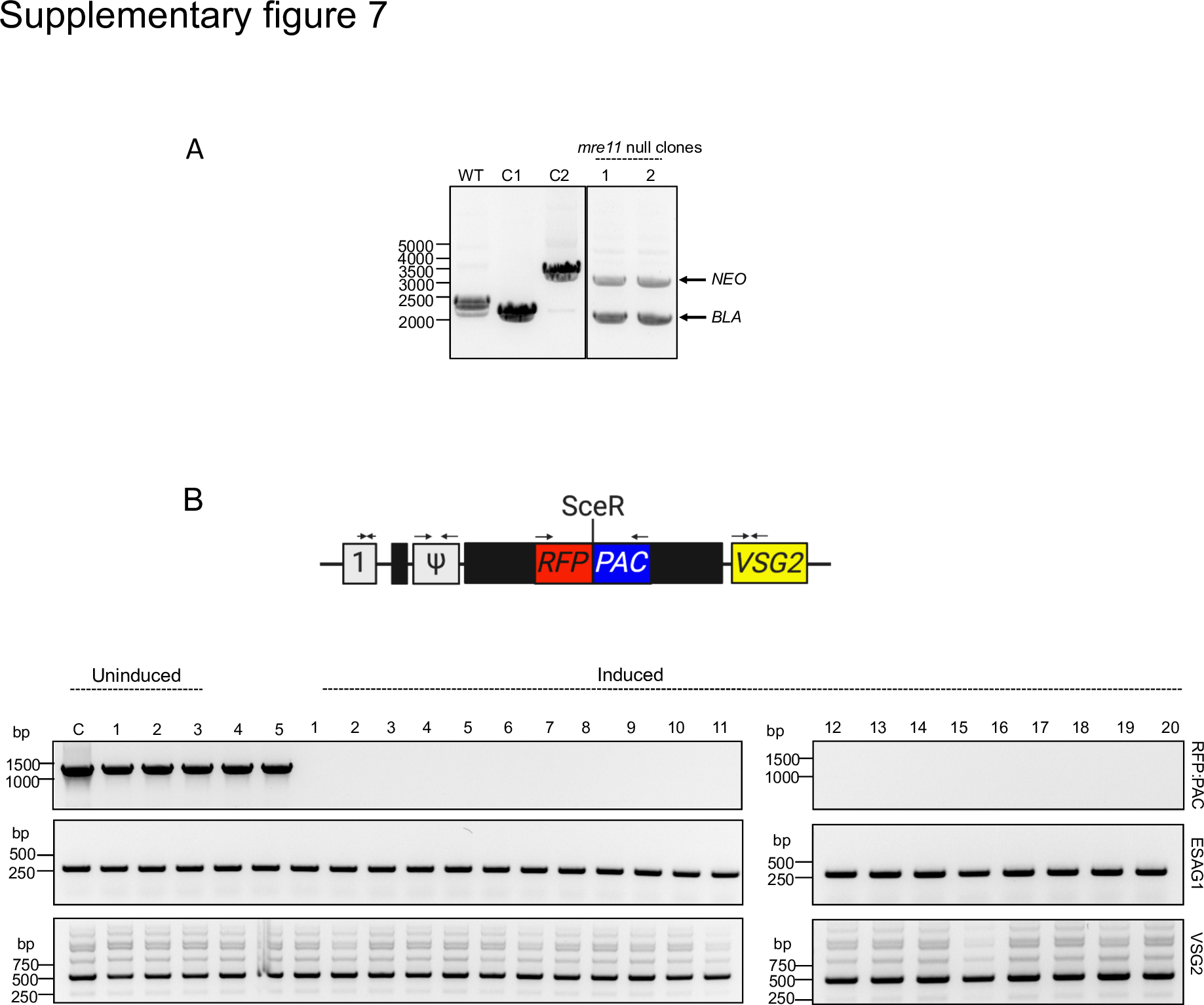

Supplement: S7 Fig — (A) PCR assay confirming mre11 double allele replacement. C1, control plasmid for BLA; C2, control plasmid for NEO. (B) Upper panel: Schematic indicates the location of the primers used. Lower panel: The PCR assays show the presence or absence of ESAG1, RFP:PAC and VSG2 following an I-SceI induced DSB. NEO, Neomycin Phosphotransferase; BLA, Blasticidin deaminase. (TIF) [file ppat.1010038.s007.tif]

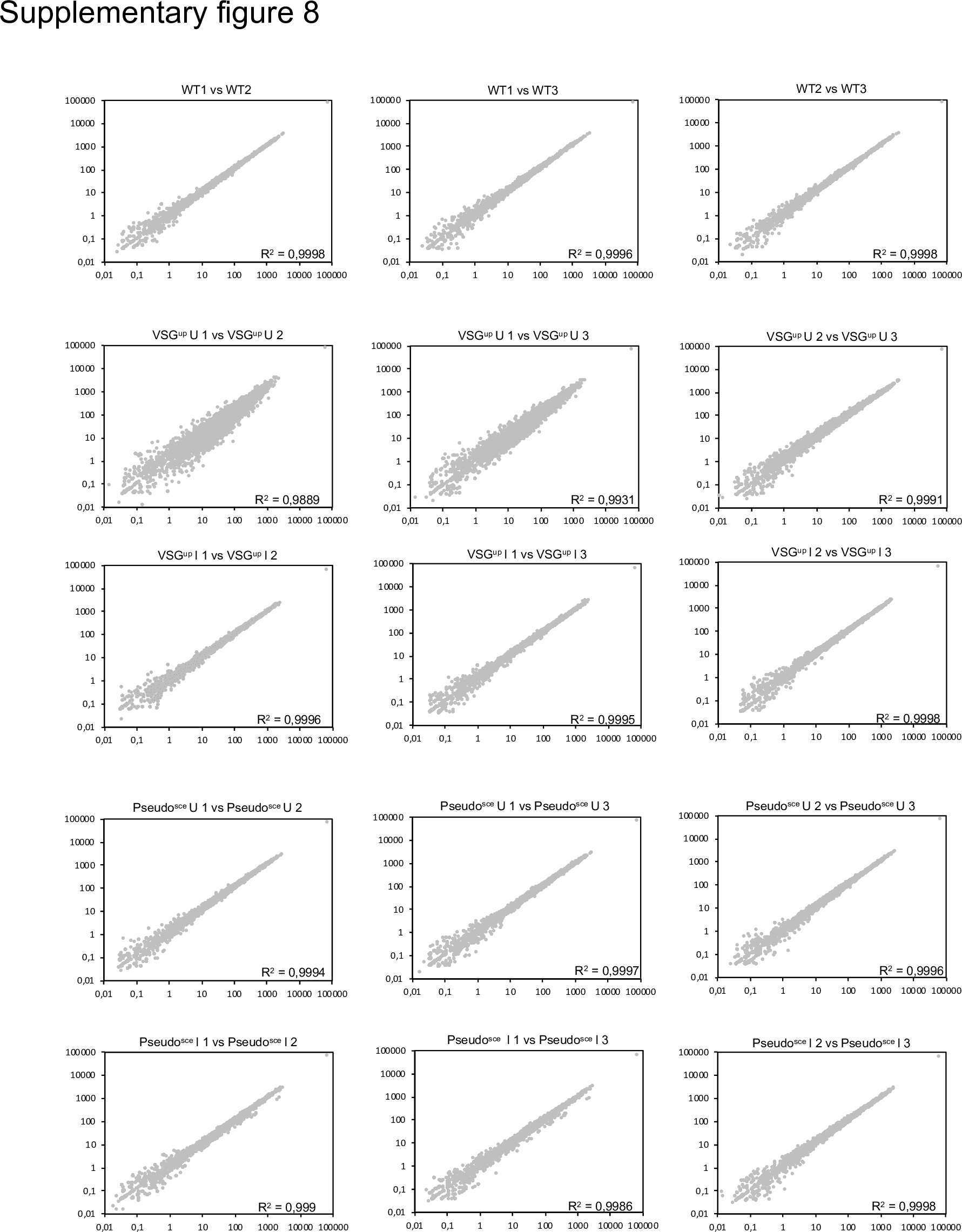

Supplement: S8 Fig — (A) The scatter plots depict pair-wise comparisons between three biological replicates of wild-type (WT) cells, VSGup uninduced (U) and induced (I) and Pseudosce uninduced (U) and induced (I). (TIF) [file ppat.1010038.s008.tif]

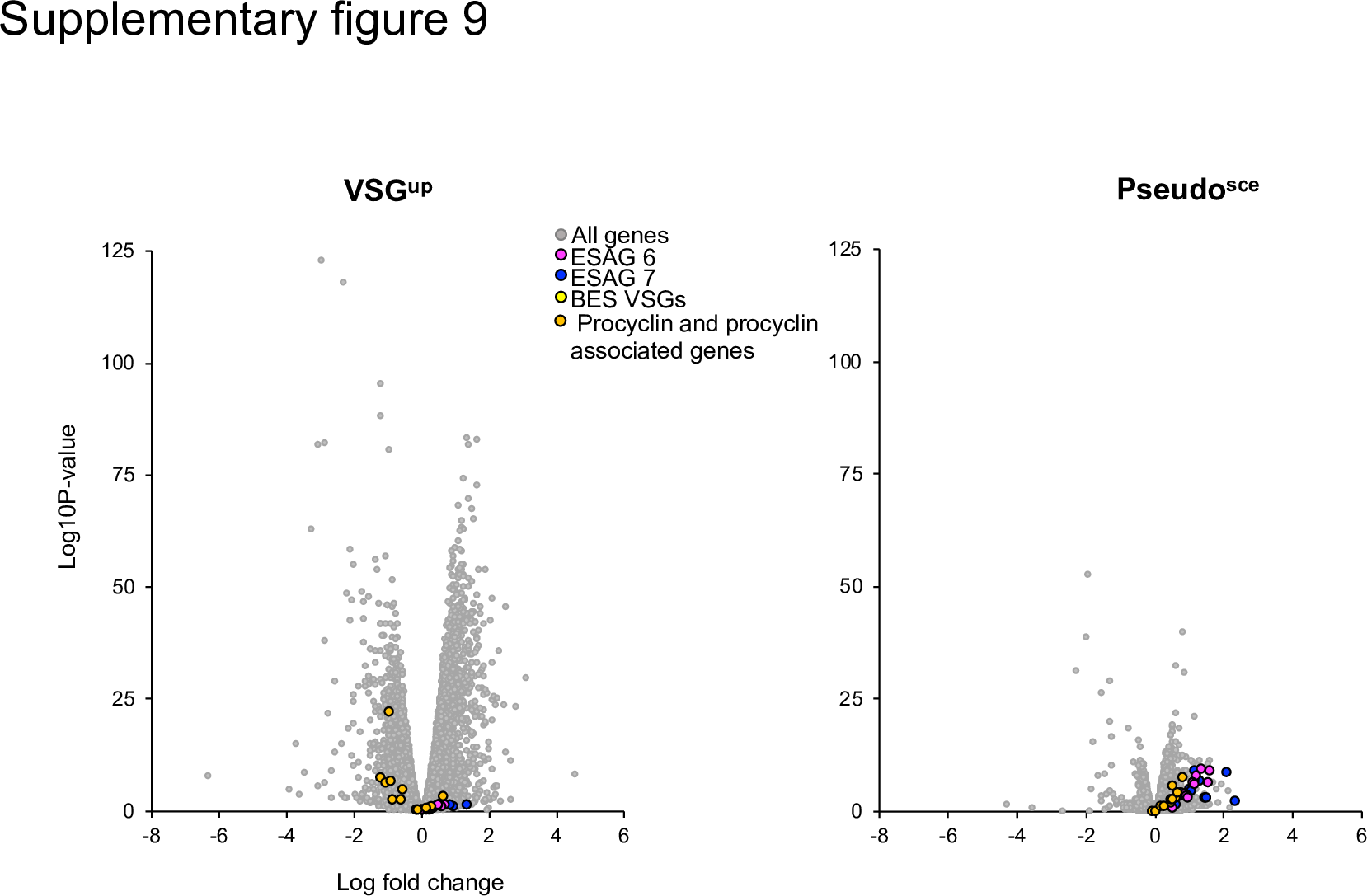

Supplement: S9 Fig — Values are averages of three independent replicates relative to wild-type controls. Yellow circles BES VSGs; pink circles, BES ESAG6; blue circles, BES ESAG7; orange circles, Procyclin and procyclin associated genes genes (All genes are included in S1 Table). (TIF) [file ppat.1010038.s009.tif]
