# Supplementary material for: DNA double strand break position leads to distinct gene expression changes and regulates VSG switching pathway choice
Source: PLoS Pathog. 2021 Nov 12;17(11):e1010038. doi: 10.1371/journal.ppat.1010038 (PMC8612549; doi:10.1371/journal.ppat.1010038)
Supplement: Attachment — Submitted filename: 118883_1_rebuttal_2190258_r0qjsm.pdf [file ppat.1010038.s012.pdf]

# Full Revision

Review COMMONS

**Manuscript number:** RC-2021-00952
**Corresponding author(s):** Lucy GLOVER

## 1. General Statements [optional]

We would like to thank the reviewers for taking the time to comment on our manuscript and for the thoughtful and helpful comments. We are very pleased that they find these data interesting and important for the trypanosome research community and beyond. This work has allowed us to map, with greater specificity, where a double strand break needs to be in order to induce antigenic variation. But more than this, we now show that the position of a break leads to distinct gene expression changes that suggest repair pathway choice is linked to where a break falls and consequently, the how VSG switching proceeds.

As the reviewers requested we have made a number of edits to the manuscript to clarify points or add details where necessary.

All changes to the manuscript are highlighted in red.

## 2. Point-by-point description of the revisions

Reviewer #1 (Evidence, reproducibility and clarity (Required)):

In this work, the Glover lab used a previously established methodology to introduce inducible double strand breaks (DSB) at the active BES, at increasingly longer distances from the VSG gene. With these cell-lines, the authors measured which breaks resulted in a VSG switch and which breaks did not result in a VSG switch. This mapping allowed the authors to confirm that a DSB immediately upstream of VSG gene triggers a VSG switch, but not in any other location. Indeed, DSB within the 70bp-repeat region, VSG pseudogene and ESAG1 were resolved by microhomology mediated repair and did not lead to expression of a new VSG.

The authors also observed that the repair of a DSB within the 70bp-repeat region was independent of RAD51, MRE11 and the timing post-DBB. Finally, in an attempt to understand how parasites, take two alternative routes (VSG switching or MMEJ), the authors characterized the transcriptome of two cell-lines 6hr post DSB-induction: VSGup (which undergoes VSG switching) and PseudoSCE (which does not undergo VSG switching). They found that the two cell-lines presented significant differences of gene expression, suggesting different reactions to a DSB. While in VSGup cell-line, the authors found an upregulation of genes involved in DNA damage and DNA repair, in PseudoSCE the most noticeable alteration is an upregulation of previously silent BESs.

Experiments were carefully controlled and the conclusions are convincing. The data and the methods are presented in such a way that they can be reproduced. Number of replicates is not always easy to find: I suggest to include this information in figure legends. On two occasions, the results are overinterpreted, but this could be easily fixed. See details below.

We thank this review for the helpful comments, and appreciate that they found our results convincing. We have endeavored to make all the changes and clarifications suggested.

**Major comments:**

1.
Figure 4A-B. The authors observe a correlation between low VSG transcript levels and the outcome of DSB. This correlation was interpreted as the outcome of the activated repair mechanism and subsequent VSG switching were influenced by whether or not VSG transcription was inhibited. A causality between VSG transcription and the outcome of DSB repair was not established. It could be the opposite: once a given mechanism of DSB repair was activated, VSG is downregulated or not. This point should be discussed more carefully.

We agree that there are alternative conclusions that can be drawn from this data. To this end we have adjusted the following statements.

In line 371 we have removed the line; "but rather whether or not *VSG* transcription was inhibited." And added 'However, this suggests that inhibition of *VSG2* transcription for over 12 hours does have an impact on VSG switching.

In the discussion we have added the following statement at line 486-89 "The reduction in *VSG2* expression could be interpreted one of two ways. Either DSB repair pathway choice results in downregulation of transcription - i.e. commitment to homology-based repair results in the formation of secondary structure that's inhibit transcription, or reduction in *VSG2* transcript triggers specific repair pathway choice."

2.
Figure 4-5. The authors observe an increase of BES-genes in PseudoSCE and they conclude that this partial activation of silent BES promoters could act as a backup should repair damage the integrity of the BES. While this is an exciting model, it would be important to show that the upregulation of silent BES is specific. In the Discussion, the authors mention that EP1 Procyclin was not found to be upregulated, suggesting that increased transcription is specific to silent BESs. To further strengthen this conclusion, the authors could analyze the rRNA genes from their transcriptome data. I understand single genes cannot be analyzed, but the authors could test whether there is a net increase in rRNA Pol I transcription or not. An alternative approach

(but more laborious) would be to perform RNAseq in a cell-line where a specific DSB is introduced in a non-BES location and test if, in this condition, there is no upregulation of silent BESs.

The RNA-seq analysis was carried out using polyadenylated enriched transcripts, and we therefore cannot look at rRNA. What we have therefore done is look at all procyclin and procyclin associated genes (PAG) and added this data in Supplementary figure 9. We have added this date into the supplementary figures as some PAG genes are related to ESAG6 and 7. But, although we see a small change in the transcript levels in the procyclin and PAG genes it is not equivalent to that see in BES linked ESAG6 and ESAG7 genes. We therefore believe that our findings stand and that we see a specific upregulation of BES linked ESAG6 and 7 genes following a DSB in the Pseudo$^{sce}$ cell line.

We have added the following statement:

Line 401-406: We included in our analysis other RNA polymerase I transcribed genes, here *Procyclin* and *procyclin associated genes* (*PAG*s). We did not see any significant change in the expression of this cohort of genes in the VSG$^{up}$ cell line and only a small shift in the Pseudo$^{sce}$ cell line. It has been reported that some *PAG* genes are closely related to ESAG6 and 7. This analysis confirms our findings that a break in the *Pseudo* gene leads to deprepression of BES linked *ESAG6* and *7*.

**Minor comments:**

3.
Figure 3C was not cited.

We apologize for this omission and have now included Figure 3 C in the text at line 341 – 346: ‘Taking all the data into account, we assessed the repair pathway choice in these cell lines. Both the Pseudo$^{sce}$ and 70int$^{sce}$ cells lines use MMEJ to repair at these positions, whereas in the VSG$^{up}$ cell line a DSB is repaired by homology-based repair, as has been previously reported [13,37] (Fig 3C). In contrast, in the ESAG1$^{sce}$ cell line, although repair was predominantly via MMEJ but 25 % of the cells were able to repair by homology-based repair (Fig 3C).’

4.
Figure legends should provide details on the number of experiments and biological replicates, and type of statistical test used.

We have ensured that all the information is found in both the materials and methods and the figure legends, and where counts performed the number or researchers who performed them. Line 146, 150-152 - Materials and methods, Figure legend 1, 2, 3, 5

Reviewer #1 (Significance (Required)):

Switching of VSGs is critical for antigenic variation. In African trypanosomes, the most frequently used mechanism of VSG switching relies on homologous recombination and it has been previously shown that double strand breaks (DSB) close to VSG gene can trigger a switch (Horn and Papavasilou's labs). However, it remained unclear if the exact position of the DSB (and in particular a break in the 70bp-repeats) influenced VSG switching. The authors confirmed that a break immediately upstream of VSG gene leads to switching, but a break further upstream doesn't, namely in the 70bp-repeat arrays. This incremental knowledge is important for the antigenic variation community because it shows that a break in the 70bp-repeat arrays is repaired by MMEJ, independently of RAD51 and MRE11. This adds a new piece of knowledge to the still elusive role of 70bp-repeats in antigenic variation.

The authors also characterised the transcriptome of parasites undergoing VSG switching or MMEJ. The descriptive list of differentially expressed genes awaits future experiments to identify the key factors that trigger and/or sustain the two mechanisms.

Expertise of this Reviewer - Trypanosomes; host-parasite interactions; VSG gene regulation.

**Referee Cross-commenting**

I strongly agree with Points 2 and 3 of Reviewer #2.

Reviewer #2 (Evidence, reproducibility and clarity (Required)):

**Summary:**

Here Thivole et al. investigate the effect of double strand break location on DNA repair and VSG switching in T. brucei. Using a collection of cell lines with an I-SceI recognition site and an inducible I-SceI endonuclease integrated into the genome, they demonstrate that only breaks immediately upstream of the VSG result in high-frequency switching. Using RNA-seq they show that a unique set of genes are upregulated in response to VSG-adjacent breaks compared to those within or upstream of the BES 70-bp repeats. They suggest that homologous recombination predominates after a VSG-adjacent break while breaks in other locations in the BES are repaired primarily using microhomology-mediated end joining.

**Major comments:**

- Are the key conclusions convincing?

The key conclusions are convincing, but I do think some claims could be qualified or clarified.

- Should the authors qualify some of their claims as preliminary or speculative, or remove them altogether?

5.
This is an unavoidable caveat as far as I can tell, but the fact that a break "within" the 70bp repeats is actually a break within an exogenous sequence placed within the repeats seems important to acknowledge. That RFP/PAC sequence seems like it could plausibly disrupt homology or some other sequence feature that would typically be used in repair. Similarly, should it be acknowledged that the VSGup line which has a unique phenotype among the lines also lacks the RFP sequence present in the other cell lines? It seems unlikely that the lack of this RFP sequence would be responsible for the patterns observed, but it probably should be acknowledged. If there is a good reason this isn't an issue, the authors should explain that in the text.

We agree that this is an unavoidable caveat and are happy to acknowledge the manner in which the break has been introduced into the difference positions. To this end we have added the following text:

Line 227: “integrated an RFP:PAC fusion cassette containing the I-SceI recognition site into the major block of 70-bp repeats”

Line 233: “in both cases using an RFP:PAC fusion cassette containing the I-SceI recognition site. An RFP:PAC fusion cassette containing the I-SceI recognition site has been used to characterise both homology-based and microhomology based repair pathways[37,49-51] and we do not believe that integration of the cassette per se would disrupt a specific repair pathway or influence repair pathway choice.”

Line 246: “In this cell line, the I-SceI recognition site is on a construct that contains the PAC cassette only.”

6.
The discussion of interruption of VSG transcription in the VSGup line (figure 4B) is confusing to me. It seems to me that it would be impossible to distinguish between decreased transcription/transcript levels and the beginning of switching, which would result in a decrease in VSG2 transcript (since switching is happening frequently in these cells and could be detected at the RNA level before switching is detected on the cell surface). The idea that the break could result in a lowering of transcript levels makes sense but I'm not sure the QPCR data show that definitively. Perhaps an assessment of total VSG could clear this up, rather than VSG2? Is that what is being shown in the lower panel of Fig 5A?

As suggested by reviewer 4, long term transcription inhibition may go further to explain the potential link between this and VSG switching. We have added a statement to this effect at line 371-2. See also point 29. Figure 5A is showing all BES linked VSG gene.

7.
Line 298 - there is a description of MMEJ scars detected by PCR, indicating repair by MMEJ, but these sequences are not shown. This seems like data that ought to be included somewhere, at least in a supplement.

We have added in an example of the MMEJ scar seen in the repaired clones into supplementary figure 2. And at line 311: “We sequenced these PCR products and in 13 clones we could detect MMEJ scars (Supplementary Fig 2C).”

8.
Line 416 - "where MMEJ dominates as the major form of repair, silent BES promoters are partially activated as a means to facilitate rapid in situ switching should repair be deleterious to the cell." I love this idea, but I might not state that this is what has been shown. This sounds more like a model that could explain the experimental results.

We agree that this is a model and so have added at line 442: “We propose a model where MMEJ dominates as the major form of repair, silent BES promoters are partially activated…”

9.
Without more details on the analysis, I think the association with DNA repair following a break in the VSGup line should be a bit more tentative. It is not clear where these annotations came from and no statistical analysis was performed as far as I can tell (I recognize GO terms in the tryp genome are fairly incomplete-especially with respect to DNA repair-so I understand why this might have been). If these associations were manually curated I worry about the potential for the introduction of bias and would acknowledge that the analysis is a bit qualitative. If it was done more systematically then this just needs to be described clearly in the text somewhere.

This analysis was done using tritryp annotations only, we did not do any blast searches or manual curation - we avoided doing this specifically, as the reviewer states, as this could introduce bias. We have added the following statement to make this clear in the text and we have stated that it is a qualitative analysis.

Line 409 ‘Using TritrypDB, we performed a qualitative assessment of these genes based on the Tritryp curations only.”

- Would additional experiments be essential to support the claims of the paper? Request additional experiments only where necessary for the paper as it is, and do not ask authors to open new lines of experimentation.
I do not think additional experiments/analyses are essential if the language reflects relevant caveats/limitations

- Are the data and the methods presented in such a way that they can be reproduced?

10.
There were some aspects of the RNA-seq analysis I wasn't sure about. The DNA repair annotation was not clear to me (where the annotations came from). I also wondered about how read counts were generated. The reason this strikes me as important, besides reproducibility, is the issue of multiply-mapping reads. I am fairly sure that bowtie2 reports multiply-mapping reads under the parameters used, which can complicate quantification of VSGs and ESAGS which have a lot of sequence similarity and result in a lot of non-uniquely-mapping reads. Also, EdgeR is designed to be used only with uniquely mapping reads. Thus, only uniquely mapping reads should have been included in the counts used for analysis. More detail on this analysis would improve the reproducibility of the analysis and shore up the claims resulting from the RNA-seq data.

For the DNA repair annotation please see point 9, we have also added in the text that this is a qualitative analysis.

It is our understanding that Bowtie in the default mode search for multiple alignments, but only reports a single best one. We have added to the materials and methods to use Bowtie in the default mode. – Line 155. According to the EdgeR users guide, it does not require uniquely mapped reads.
(https://bioconductor.org/packages/release/bioc/vignettes/edgeR/inst/doc/edgeRUsersGuide.pdf)

11.
Figure 3C is not mentioned in the text; I'm not sure where these numbers come from. Also, the 3-D format makes the graph a little harder to read than necessary, if that figure is meant to be included.

We apologize for this omission. Please see point 3 for the corrections.
We have changed the graph, it is no longer 3D

**Minor comments:**

12.
Line 252 paragraph - the description of G2 checkpoint data is a bit confusing. It's not clear that "(9 hours)" means "peaked at 9 hours" and took me a while to sort out. I don't find mention of those peak timepoints to be particularly informative, either, given the size of the error bars in that dataset. I think the "(x hours)" part could be removed to improve readability.

We have rewritten this part for clarity and removed the hours and fold change values. The statement now reads

Line 264: "In the ESAG1$^{sce}$, Pseudo$^{sce}$, 70int$^{sce}$ and VSG$^{up}$ cells lines we saw an increase in cells in G2 between 6 to 12 hours following induction (Fig 1D). For the γH2A focal accumulation, we saw an increase in the number of foci at 12 hours post DSB induction in the ESAG1$^{sce}$, Pseudo$^{sce}$, 70int$^{sce}$ and VSG$^{up}$ respectively (Fig 1E)."

- I also found the description of clones in lines 261-307 a little hard to follow. Perhaps some of this data could be summarized into a table or otherwise streamlined?

We have rewritten parts of this section for clarity. See lines 277-307

Reviewer #2 (Significance (Required)):

DNA repair within the VSG BES is critical to the process of antigenic variation in T brucei, and this study analyzes the effect of break location on repair outcomes and VSG switching. It was unknown whether the position of a DNA break influenced switching frequency or outcomes and this work shows that breaks in specific areas favor switching and suggest that different mechanisms of repair are used in each location. Thus, this work begins to address an important question in the study of antigenic variation and DNA repair in T. brucei. This paper would be of interest to investigators interested in antigenic variation (either in T. brucei or pathogens with similar systems of antigenic variation) as well as those interested in DNA repair more generally. My expertise is in antigenic variation in T. brucei and specifically using bioinformatics approaches to study the process.

Reviewer #4 (Evidence, reproducibility and clarity (Required)):

The authors have systematically screened the effect of DSB position on VSG recombination/switching, which is important for defining the regions/sequences within the BES that are required for this process. Their data suggests that for switching to occur a DSB needs to happen in close proximity to the VSG gene with breaks further away from the VSG being repaired without switching. Plus they demonstrated the position of the break influences the repair mechanism and transcriptional changes.

Overall the data presented supports the conclusions the authors draw, though in my detailed comments I have pointed out a couple of areas where these need some modification. In places I found the figures difficult to follow and the legends where light on detail and these areas need some attention. In my view the presented experiments are sufficient and I don't think any additional experiments are required. The methodology is clear and the statistical analysis appears to be fine.

**Detailed comments**

13.
Line 38 - The phrase 'Underlying this process' makes it sound like all switches are due to gene switching in and out of BES, which is not the case.

We have changed this to:
Line 38: "This process is facilitated by"

14.
Line 175 - should read 'lengths between 500 bp to 2 kb'

Thank you – this has been corrected.

15.
Line 188 - qPCR

Thank you – corrected.

16
Throughout the manuscript the figure legends were light on detail and need to be improved to help the reader understand the figures more easily. For example in Supp fig. 1, it says Southern blot with no more details - the information is in the methods but including the probe/enzymes would be helpful.

We apologize to the reviewers for the lack of detail and have now rectified this. Please see point 4.

17.
Figure 1D/E - what do the error bars show? If there were only 2 inductions would be better just to show the individual data points.

The error bars are the SD of two inductions and counts does by two researchers.

18.
Line 238 - the authors dive straight into the results and did not mention they induced the cells and it took me a moment to work out what had happened.

To clarify this, we have added the following statement at line 247: "To assess the effect of a DSB in these cell lines, we induced I-SceI using tetracycline."

19.
Line 254 - add 'following induction'

Corrected as requested – see line 264

20.
Line 265 - within the sequence

Thank you, this has been corrected.

21.
I found supp figure 2 and the text describing it a bit confusing. For the amplification of RFP:PAC there was a weak band clone 1 that was approximately the correct size and not mentioned. In the RFP:PAC clone 13-20 panel there are only 7 lanes not 8 so not sure what is happening here.

We apologize for this error and are very grateful to the reviewer for pointing this out. For the RFP:PAC gel set, clones 13-19 were on one gel and 20-24 on a second. We have gone back to the original images and remade this panel.

For the weak band in lane 1, this clone was puromycin sensitive and no cells grew out of the treated culture suggesting they had all been cut. This band then either represents a background band or an MMEJ event that resulted in a small deletion that is not detectable. We have added this into the text at line 301-305

22.
Line 293 - puromycin resistant clone 23 - is it not 17?

This has been corrected now line 300.

23.
Supp figure 2 legend has A-C but the figure is only A-B.

C has now been deleted.

24.
Line 298/299 is this referring to Figure 3C? If not the data should be included somewhere. Is there any reason for Figure 3C to be a 3D graph?

Figure 3C is no longer in 3D and see point 3 for the associated text.

25.
Line 305 - clone 12 appears to have retained VSG2 - does this suggest this region has been moved to another bit of the genome or a hybrid VSG is being expressed?

Given the weakness of the band we think that this is a background band.

26.
Supplementary figure 6 mentions a western blot but I can't see that in the figure.

This was an error and the reference to a western blot has been removed.

27.
Supp figure 7 - can you include the cell line name that panel B is referring to.

We have changed the figure title to Generation of *mre11* null in the 70int$^{sce}$ cell line so reflect the parental cell line.

28.
Line 324 - Leishmania in italics

Thank you, this has been corrected.

29.
Line 352-4 - I agree there seems to be little connection between timing of cleavage and repair/switching, though there is only one cell line ESAG1sce that has a dramatically different cleavage kinetics so firm conclusions are tricky on this point. The evidence for switching based on VSG transcription inhibition is not quite right as for the first 12 hours the drop in VSG transcription for 70intsce is greater or similar to VSGup and these cell lines have different switching outcomes. From these data what appears important is a longer term transcription inhibition of over 12 hours.

We agree with the reviewer that long term transcription inhibition will be important and so have added a statement to this effect at line 371-3: “not influenced by the timing of I-SceI cleavage, but suggests that inhibition of *VSG2* transcription for over 12 hours does have an impact on VSG switching.“

30.
For figure 4 can you highlight VSG2 transcript, I assume for VSGup it is the red dot towards the top left of the volcano plot.

We have added VSG2 (Figure 4C white dot) to the plots in the middle panel. Because we are looking at fold change at 6 hours we don’t expect to see large change in the amount of VSG2 transcript.

31.
Line 368 - 11.32

Thank you - Corrected

32.
Line 368-9 - I struggled to follow the data in the bracket is this comparing the changes between the VSGup and Pseudosce cell lines?

We can see how this could be confusing. This is reporting the range in the fold change and log10 between all BES linked ESAG7 and ESAG6. We have changed the statement for clarity. It now reads:

Line 386: (between the 16 x *ESAG7* genes we see a range in the fold change (FC) from 0.48 to 2.3 and $Log_{10}$ from 1.64 to 10.64; between the 14 x *ESAG6* genes we see a range in the FC from 1.62 to 0.46 and $Log_{10}$ from 0.95 to 11.32)

33.
Line 433 - response is dampened

Thank you – this has been corrected, now line 458

34.
Line 482 - specific to where

We have clarified this statement to now reads :

Line 511: and specific to where a DNA break is repaired by MMEJ.

Reviewer #4 (Significance (Required)):

DSBs have long been known to be important for triggering VSG switching in trypanosomes but this is the first study to pin down where this DSB needs to occur for VSG switching. The authors found that the DSB needs to occur in close proximity to the VSG and this is an important insight into the process that underpins the ability of the parasite to persist in its mammalian host. Moreover, the authors show that the position of the DSB also influences subsequent transcriptional and repair responses, which presumably are connected to whether a switch ultimately occurs.

This is an important and interesting study, which will of course be of great interest to trypanosome biologists be also to the wider antigenic variation community including malaria researchers as antigenic variation is a commonly used immune evasion strategy used by pathogens.

I am a molecular cell biologist who specialises in trypanosome and leishmania cell biology.

# Full Revision
